# Supplementary material for: Assembly of the 81.6 Mb centromere of pea chromosome 6 elucidates the structure and evolution of metapolycentric chromosomes
Source: PLoS Genet. 2023 Feb 3;19(2):e1010633. doi: 10.1371/journal.pgen.1010633 (PMC10027222; doi:10.1371/journal.pgen.1010633)
Supplement: S1 Fig — The nanopore “seed” reads used to initiate CEN6 scaffolding were selected based on the presence of sequences of genetic markers from the nonrecombining region of linkage group LGII or the sequences of CEN6-specific satellite repeats. (A) The positions of genetic marker sequences in the assembly (x-axis) compared with their positions on the genetic map. Markers highlighted in green were physically localized on chromosomes (panel F). (B) The position of the primary constriction in the assembly. Arrows below the scale indicate the 5.2 Mb tandem duplication, and the arrowhead indicates the position of a single gap in the assembly. (C) Positions of the satDNA arrays, with the three CEN6-specific families marked with asterisks. (D) Regions of the assembly that were scaffolded with nanopore reads or constructed from HiFi contigs are shown by horizontal bars. Dots mark gaps in nanopore scaffolds corresponding to satDNA arrays that were filled using HiFi contigs. (E-F) Examples of assembly verification using FISH. (E) Localization of selected satellite repeats on pachytene chromosomes. Note that smaller FabTR-10 signals are not visible due to the short exposure time. (F) Sequences of genetic markers (green) detected on metaphase chromosome 6 using the highly-sensitive single-copy FISH protocol. Satellite PisTR-B (red) was used to discriminate chromosomes within the pea karyotype. (PDF) [file pgen.1010633.s001.pdf]

Supporting Information Appendix

Fig. S1

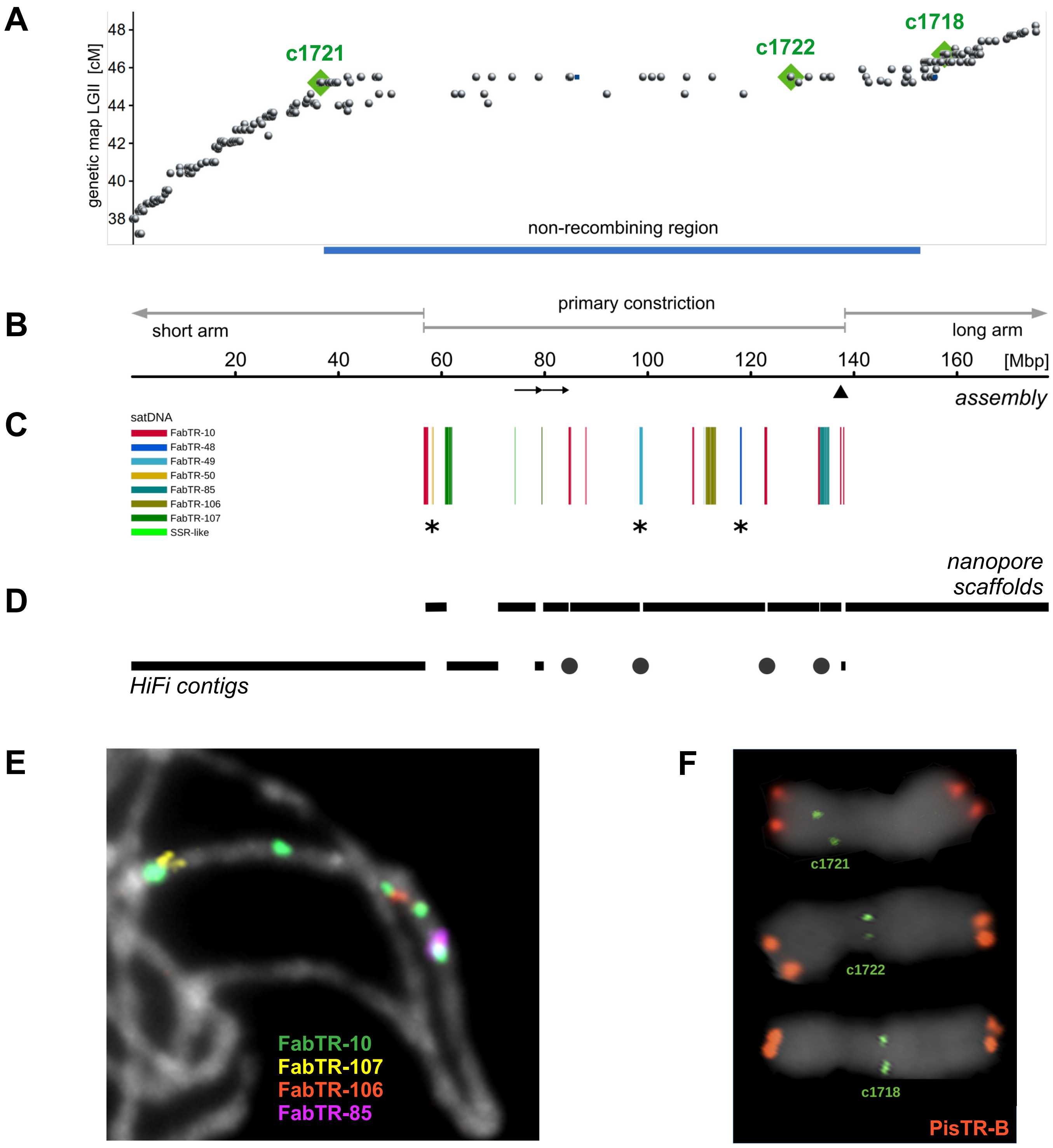

S1 Fig. Assembly construction and verification using genetically and physically localized markers. The nanopore "seed" reads used to initiate CEN6 scaffolding were selected based on the presence of sequences of genetic markers from the nonrecombining region of linkage group LGII or the sequences of CEN6-specific satellite repeats. (**A**) The positions of genetic marker sequences in the assembly (x-axis) compared with their positions on the genetic map. Markers highlighted in green were physically localized on chromosomes (panel F). (**B**) The position of the primary constriction in the assembly. Arrows below the scale indicate the 5.2 Mb tandem duplication, and the arrowhead indicates the position of a single gap in the assembly. (**C**) Positions of the satDNA arrays, with the three CEN6-specific families marked with asterisks. (**D**) Regions of the assembly that were scaffolded with nanopore reads or constructed from HiFi contigs are shown by horizontal bars. Dots mark gaps in nanopore scaffolds corresponding to satDNA arrays that were filled using HiFi contigs. (**E-F**) Examples of assembly verification using FISH. (**E**) Localization of selected satellite repeats on pachytene chromosomes. Note that smaller FabTR-10 signals are not visible due to the short exposure time. (**F**) Sequences of genetic markers (green) detected on metaphase chromosome 6 using the highly-sensitive single-copy FISH protocol. Satellite PisTR-B (red) was used to discriminate chromosomes within the pea karyotype.
