# Supplementary material for: Assembly of the 81.6 Mb centromere of pea chromosome 6 elucidates the structure and evolution of metapolycentric chromosomes
Source: PLoS Genet. 2023 Feb 3;19(2):e1010633. doi: 10.1371/journal.pgen.1010633 (PMC10027222; doi:10.1371/journal.pgen.1010633)
Supplement: S2 Fig — Duplicate experiments were performed for each CENH3 gene variant using either two different antibodies (P22 and P43 for CENH3-1) or one antibody (P23 for CENH3-2). The number of reads mapped onto the assembly was presented either as a ratio of ChIP-seq reads to genomic (input DNA) reads (lanes "ratio") or as regions of significant ChIP-seq enrichment identified with the epic2 and macs2 programs. (A,B) Mapping of reads onto the assembly either in multilocus mode (A) or single-mapping mode (B). In (A), multiple mappings of repetitive reads were allowed. In (B), only the reads with unique hits were mapped, and repetitive reads were discarded. (PDF) [file pgen.1010633.s002.pdf]

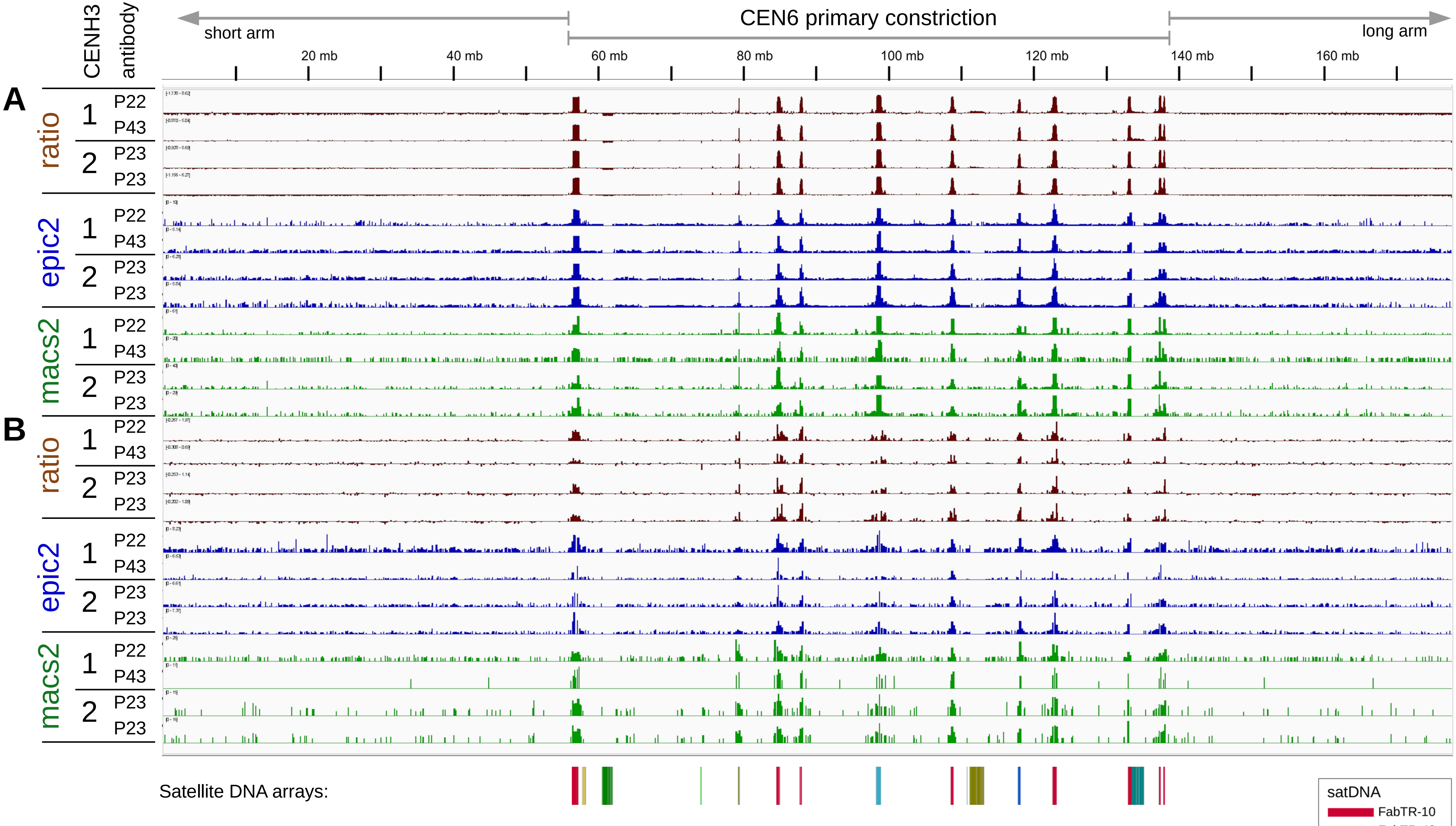

S2 Fig. Localization of centromeric chromatin by CENH3 ChIP-seq. Duplicate experiments were performed for each CENH3 gene variant using either two different antibodies (P22 and P43 for CENH3-1) or one antibody (P23 for CENH3-2). The number of reads mapped onto the assembly was presented either as a ratio of ChIP-seq reads to genomic (input DNA) reads (lanes "ratio") or as regions of significant ChIP-seq enrichment identified with the epic2 and macs2 programs. **(A-B)** Mapping of reads onto the assembly either in multilocus mode (**A**) or single-mapping mode (**B**). In (**A**), multiple mappings of repetitive reads were allowed. In (**B**), only the reads with unique hits were mapped, and repetitive reads were discarded.
