# Supplementary material for: Assembly of the 81.6 Mb centromere of pea chromosome 6 elucidates the structure and evolution of metapolycentric chromosomes
Source: PLoS Genet. 2023 Feb 3;19(2):e1010633. doi: 10.1371/journal.pgen.1010633 (PMC10027222; doi:10.1371/journal.pgen.1010633)
Supplement: S3 Fig — Per-base cytosine methylation frequencies in three sequence contexts known in plants (CpG, CHG, CHH) were obtained by analyzing Oxford Nanopore reads aligned to the assembly using DeepSignal-plant (Ni et al., 2021) [24]. (A) The plots show the fraction of aligned nanopore reads, in which cytosine was methylated at a given position. The total number of aligned nanopore reads is indicated in the "coverage" plot. The distribution of CENH3 chromatin and annotations of the major families of satDNA are shown for comparison with the methylation profiles. (B,C) Detailed examples of hypomethylated regions. Hypomethylated arrays of satDNA are marked with asterisks. (B) Sequence at the short-arm constriction junction contains CHG-hypomethylated FabTR-10, whereas the array of the same repeat within the constriction has a normal methylation level (C, marked with “x”). Short hypomethylated islands are best seen in the gene-rich region marked in (C). The CENH3 ChIP-seq track shows enrichment peaks identified by the epic2 program using multi-mapped reads generated with the P22 antibody. (D) Per-base methylation frequency distributions within specific regions or sequence types. Distributions were calculated for the entire primary constriction (“CEN”) and chromosome arm (“arms”) sequences as well as for specific satellite repeats and genes. Gene sequences occurring in the centromere (CEN) and chromosome arms were analyzed separately. Red arrowheads mark the position of peaks corresponding to hypomethylated genes. (PDF) [file pgen.1010633.s003.pdf]

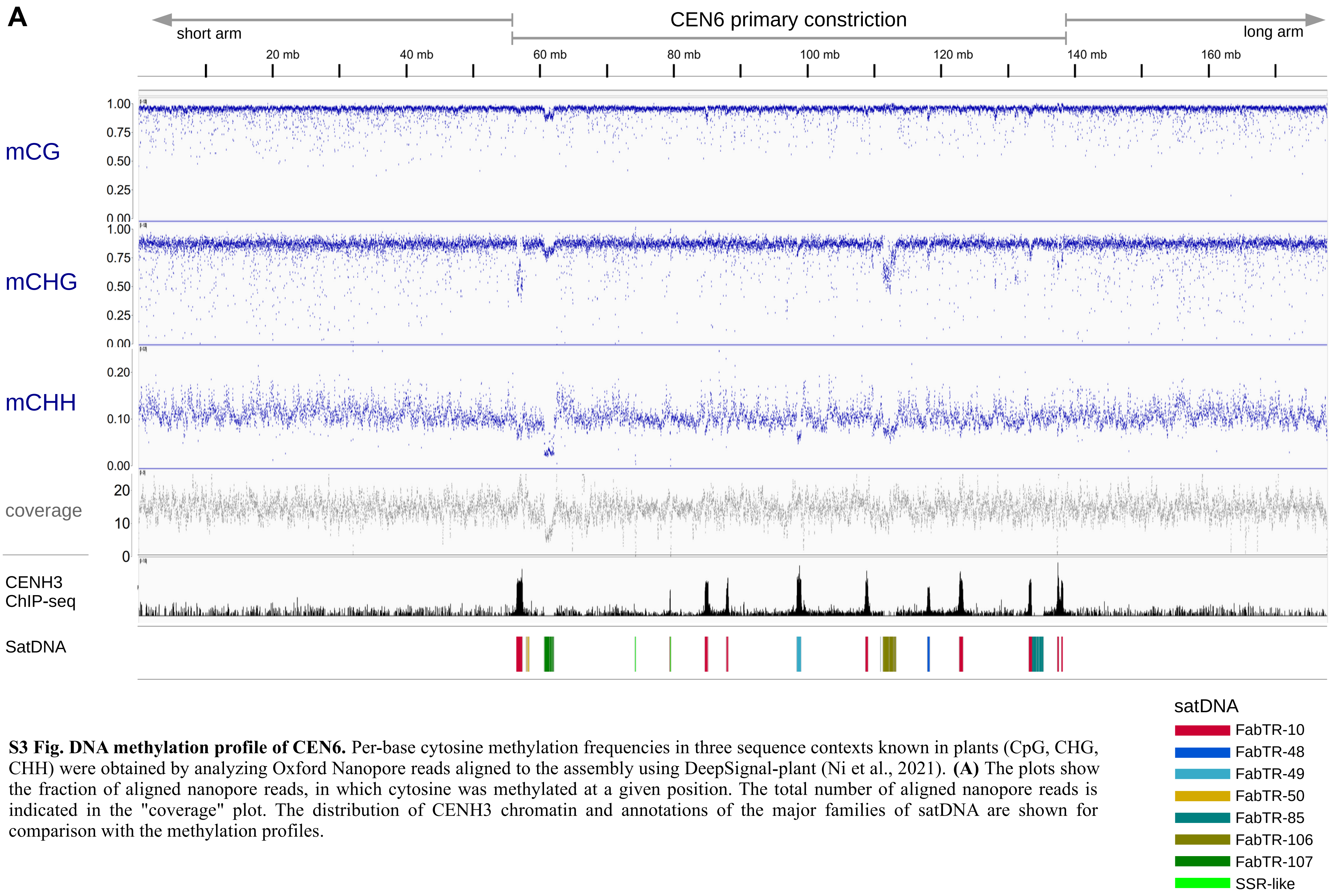

S3 Fig. DNA methylation profile of CEN6. Per-base cytosine methylation frequencies in three sequence contexts known in plants (CpG, CHG, CHH) were obtained by analyzing Oxford Nanopore reads aligned to the assembly using DeepSignal-plant (Ni et al., 2021). **(A)** The plots show the fraction of aligned nanopore reads, in which cytosine was methylated at a given position. The total number of aligned nanopore reads is indicated in the "coverage" plot. The distribution of CENH3 chromatin and annotations of the major families of satDNA are shown for comparison with the methylation profiles.

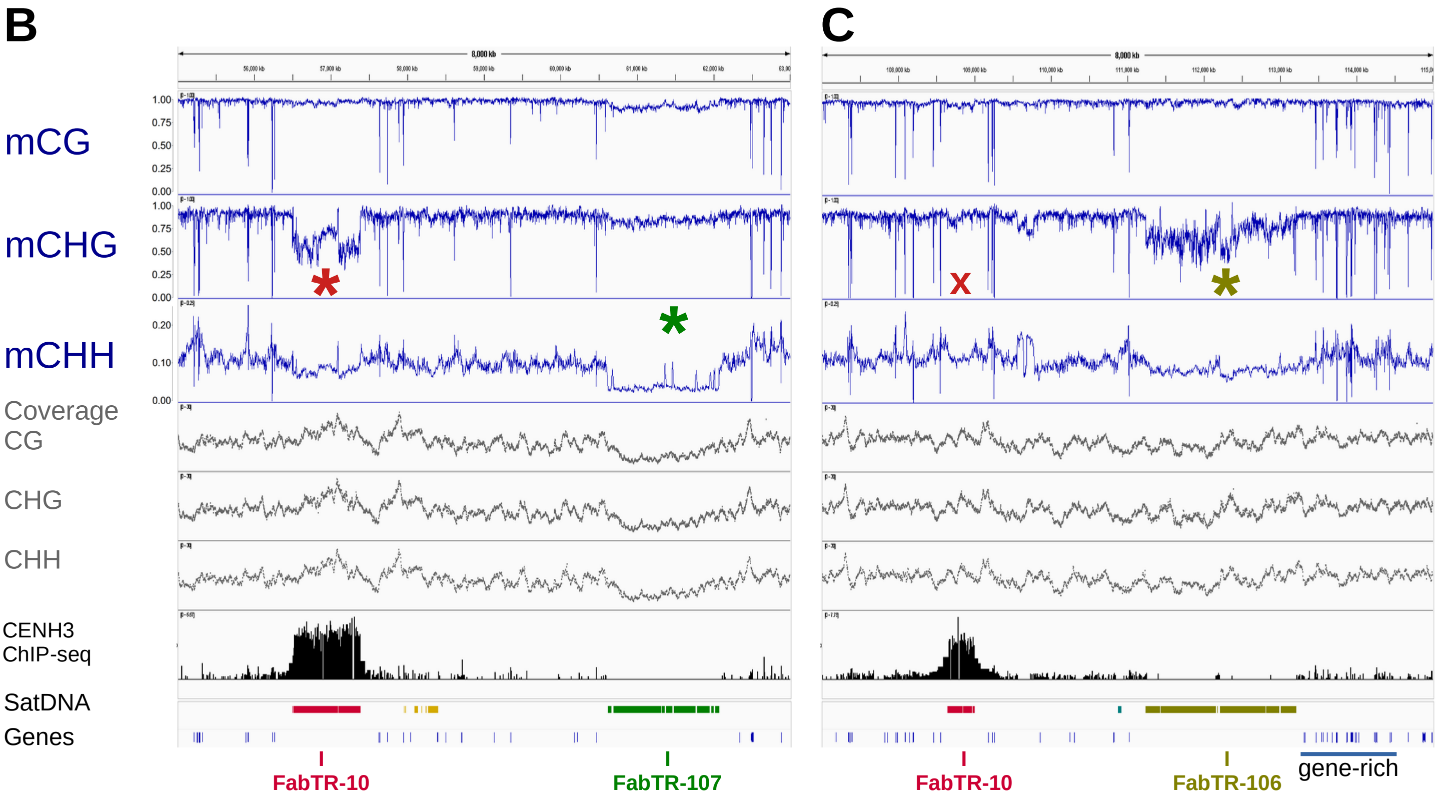

S3B,C Fig. Detailed examples of hypomethylated regions. Hypomethylated arrays of satDNA are marked with asterisks. (**B**) Sequence at the short-arm constriction junction contains CHG-hypomethylated FabTR-10, whereas the array of the same repeat within the constriction has a normal methylation level (**C**, marked with “x”). Short hypomethylated islands are best seen in the gene-rich region marked in (C). The CENH3 ChIP-seq track shows enrichment peaks identified by the epic2 program using multi-mapped reads generated with the P22 antibody.

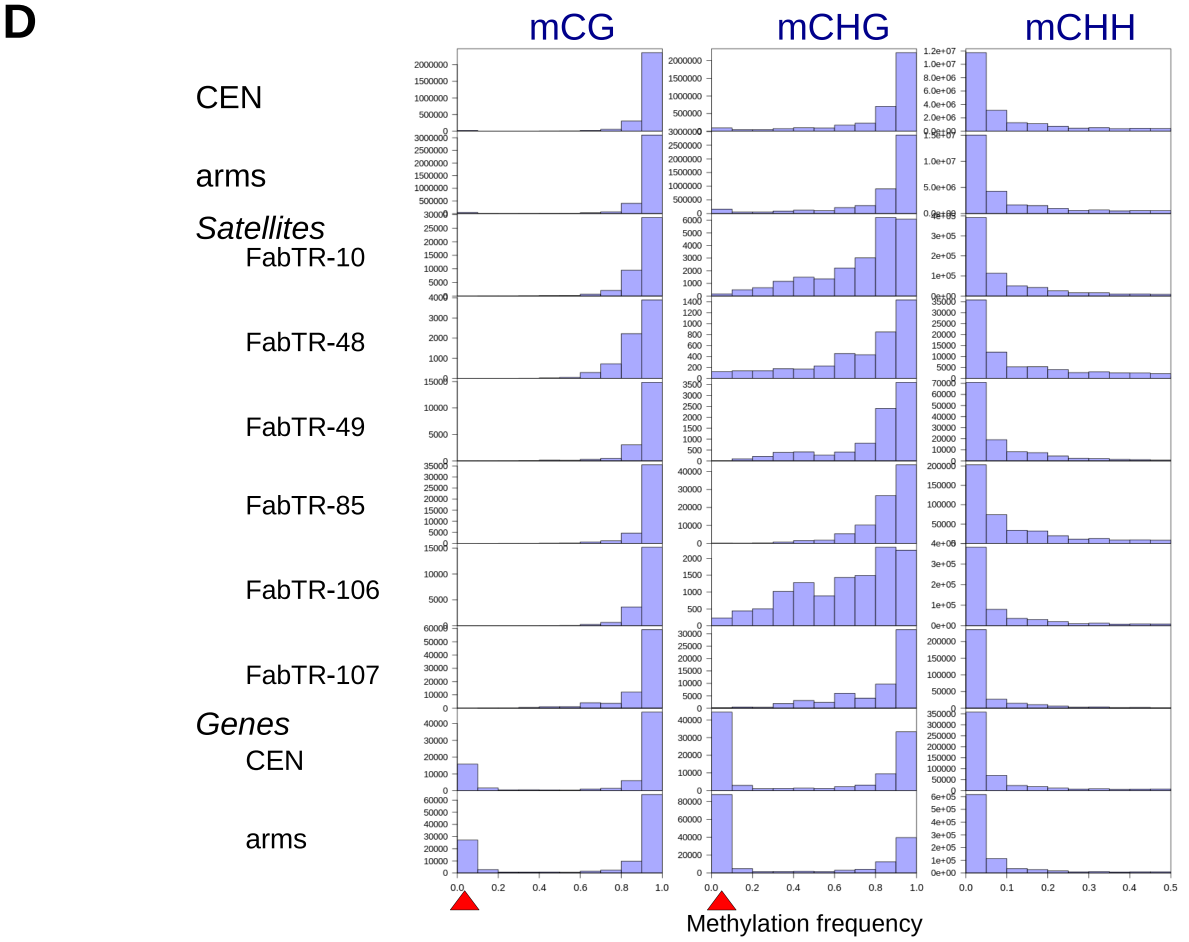

S3D Fig. Per-base methylation frequency distributions within specific regions or sequence types. Distributions were calculated for the entire primary constriction (“CEN”) and chromosome arm (“arms”) sequences as well as for specific satellite repeats and genes. Gene sequences occurring in the centromere (CEN) and chromosome arms were analyzed separately. Red arrowheads mark the position of peaks corresponding to hypomethylated genes.
