# Supplementary material for: Assembly of the 81.6 Mb centromere of pea chromosome 6 elucidates the structure and evolution of metapolycentric chromosomes
Source: PLoS Genet. 2023 Feb 3;19(2):e1010633. doi: 10.1371/journal.pgen.1010633 (PMC10027222; doi:10.1371/journal.pgen.1010633)
Supplement: S4 Fig — The data shown represent zoomed-in sections of the graphs shown in S2 and S3 Figs corresponding to loci with FabTR-10 arrays. The positions of the arrays are indicated by gray bars below the graphs and are complemented by sequence homogenization dot plots (compiled from Fig 1). (PDF) [file pgen.1010633.s004.pdf]

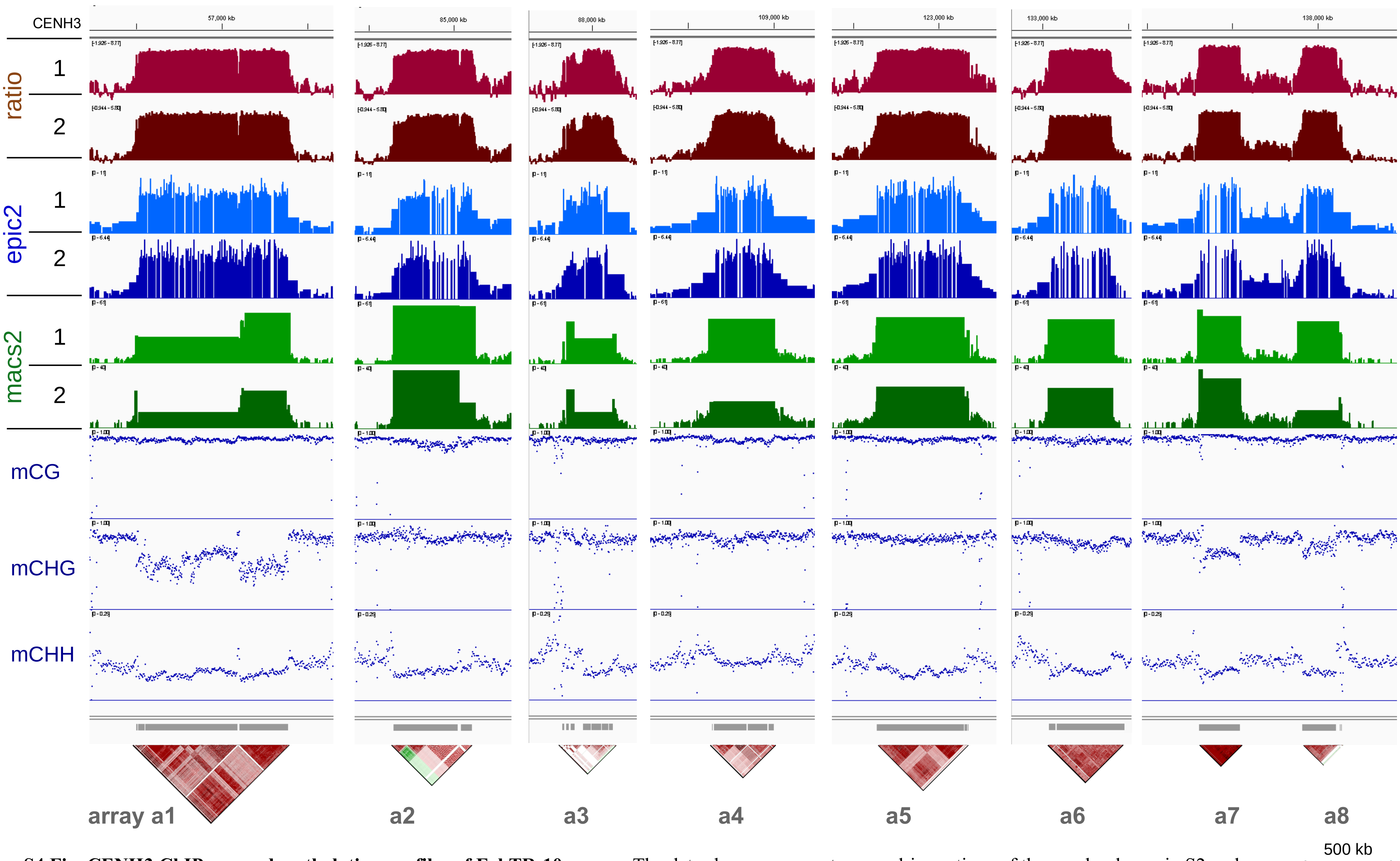

S4 Fig. CENH3 ChIP-seq and methylation profiles of FabTR-10 arrays. The data shown represent zoomed-in sections of the graphs shown in S2 and S3 Figs corresponding to loci with FabTR-10 arrays. The positions of the arrays are indicated by gray bars below the graphs and are complemented by sequence homogenization dot plots (compiled from Fig. 1).
