# Supplementary material for: Assembly of the 81.6 Mb centromere of pea chromosome 6 elucidates the structure and evolution of metapolycentric chromosomes
Source: PLoS Genet. 2023 Feb 3;19(2):e1010633. doi: 10.1371/journal.pgen.1010633 (PMC10027222; doi:10.1371/journal.pgen.1010633)
Supplement: S5 Fig — (A) Chromosome complements of selected Fabeae species hybridized with PS6-C (green) and PS6-A (red) painting probes. Bar = 10 μm. (B) Hybridization pattern of CEN6 painting probes on chromosome 6 of Pisum fulvum. Left panel: extent of the primary constriction (white bar), as revealed by the immunolabeling of CENH3 and the FISH detection of PisTR-B repeats, showing that PisTR-B is located just above the CENH3 signals. Right panel: combined FISH detection using the painting probes together with the PisTR-B probe, which was used as a reference for the end of the constriction and shows that the green PS6-C probe extends into the short arm. (PDF) [file pgen.1010633.s005.pdf]

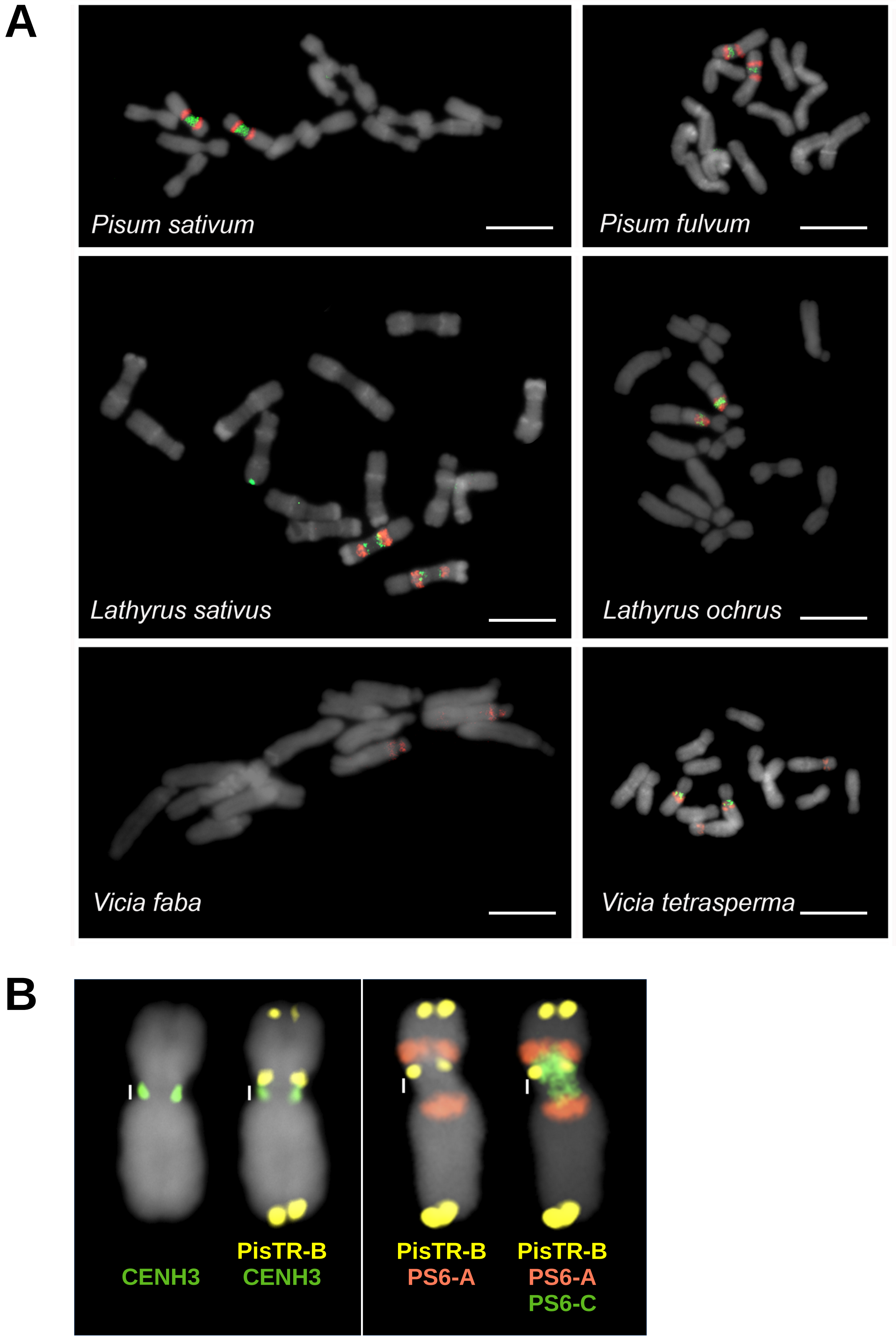

S5 Fig. FISH with CEN6 painting probes. (**A**) Chromosome complements of selected Fabeae species hybridized with PS6-C (green) and PS6-A (red) painting probes. Bar = 10 µm. **(B)** Hybridization pattern of CEN6 painting probes on chromosome 6 of *Pisum fulvum*. *Left panel:* extent of the primary constriction (white bar), as revealed by the immunolabeling of CENH3 and the FISH detection of PisTR-B repeats, showing that PisTR-B is located just above the CENH3 signals. *Right panel:* combined FISH detection using the painting probes together with the PisTR-B probe, which was used as a reference for the end of the constriction and shows that the green PS6-C probe extends into the short arm.
