# Supplementary material for: Assembly of the 81.6 Mb centromere of pea chromosome 6 elucidates the structure and evolution of metapolycentric chromosomes
Source: PLoS Genet. 2023 Feb 3;19(2):e1010633. doi: 10.1371/journal.pgen.1010633 (PMC10027222; doi:10.1371/journal.pgen.1010633)
Supplement: S6 Fig — The dot-plot shows all-to-all sequence comparison of consensus monomer sequences representing individual satDNA families. Similarities were scored within a sliding window of 100 bp and dots or lines were drawn when at least 50 matching bases were detected. A single monomer copy is compared for all families, except for families with short monomers, where three (FabTR-85) and ten (FabTR-107) concatenated monomers were used for comparison. The lengths of the consensus monomers are given in parentheses. (PDF) [file pgen.1010633.s006.pdf]

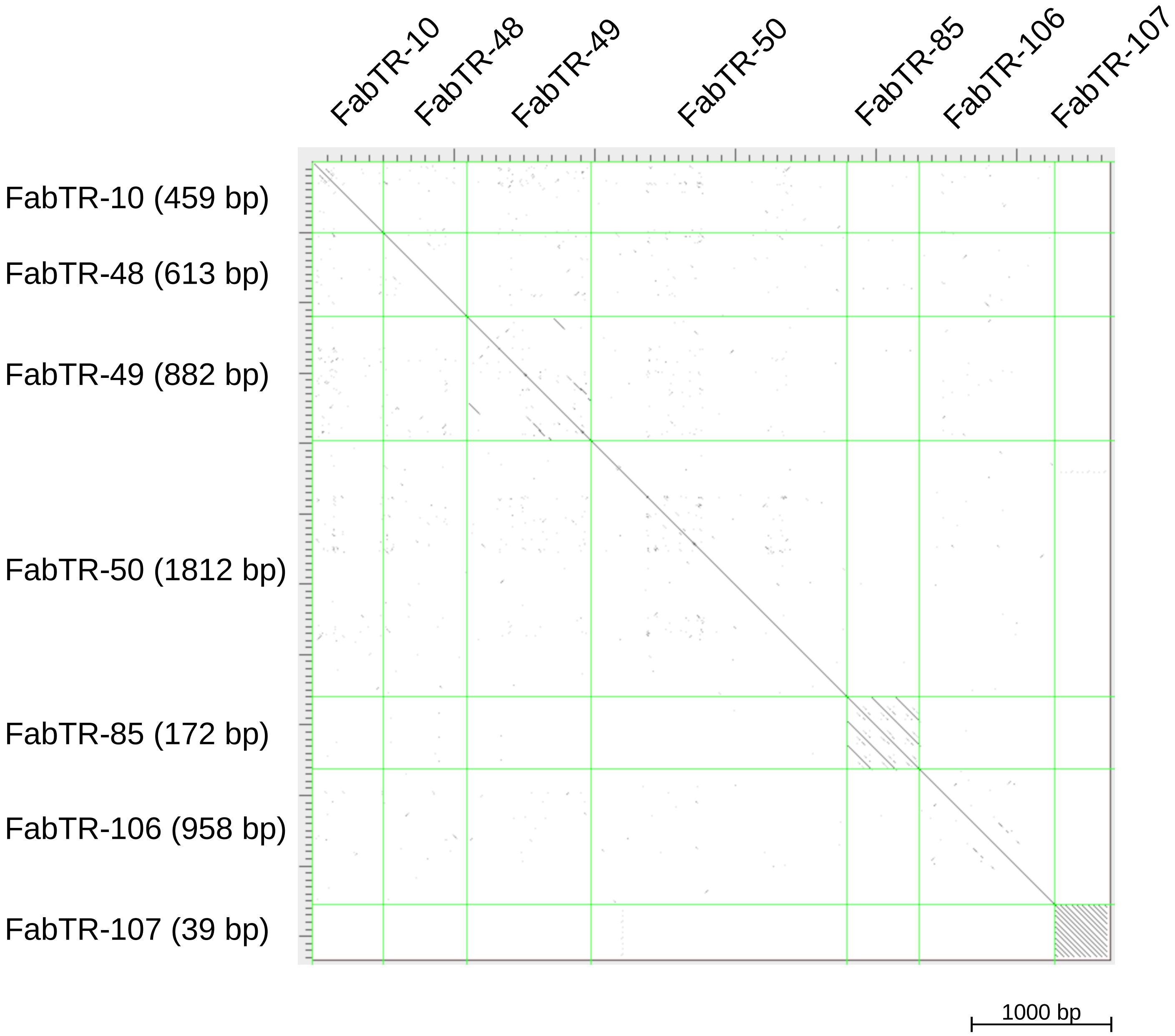

S6 Fig. Monomer lengths and sequence similarities between satellite repeat families present in CEN6. The dot-plot shows all-to-all sequence comparison of consensus monomer sequences representing individual satDNA families. Similarities were scored within a sliding window of 100 bp and dots or lines were drawn when at least 50 matching bases were detected. A single monomer copy is compared for all families, except for families with short monomers, where three (FabTR-85) and ten (FabTR-107) concatenated monomers were used for comparison. The lengths of the consensus monomers are given in parentheses.
