# Supplementary material for: Assembly of the 81.6 Mb centromere of pea chromosome 6 elucidates the structure and evolution of metapolycentric chromosomes
Source: PLoS Genet. 2023 Feb 3;19(2):e1010633. doi: 10.1371/journal.pgen.1010633 (PMC10027222; doi:10.1371/journal.pgen.1010633)
Supplement: Attachment — Submitted filename: response_to_reviewers_and_list_of_revisions_230109.pdf [file pgen.1010633.s007.pdf]

Response to the reviewers' comments and corresponding changes made to the manuscript

We are very grateful to the reviewers for their positive evaluation of our manuscript and insightful comments. We have modified the manuscript text as suggested and included additional data where requested, as specified below.

Reviewer #1: The authors deciphered the largest-ever analyzed centromere, the so-called "metapolycentromere" of the pea chromosome 6. This 81.6 Mb-long centromere was analyzed to understand the organization of such an unusual centromere type and to get insight into the molecular and evolutionary mechanisms leading to centromere-type variations. Generally, two types of centromeres exist, mono- and holocentric chromosomes, but how holocentromere evolved is still unclear. Notably, the metapolycentromere shares several features of a holocentromere. Therefore metapolycentric chromosomes provide a unique chance to study the changes associated with the transition between different centromere organizations.
I enjoyed reading this manuscript because all conclusions are based on conclusive data, and the findings are new and exciting. I have only a few comments which are listed below.

Page 2, line 40
Remove "plant", because the same applies to non-plant species

Response: Thank you for this suggestion. The modified sentence now reads: "Despite their conserved function, centromeres exhibit considerable variation…"

Page 3, line 84.
Please be more precise. The sat DNA interacts with CENH3-containing nucleosomes.

Response: Thank you for this suggestion. The modified sentence now reads: "…. satDNA is of particular interest because it is known to be a key sequence component that interacts with CENH3-containing nucleosomes in many centromeres [11]."

Page 5, line 117
The title is not understandable for a non-specialist. I would suggest (or similar) "Assembly of the polymetacentromere of pea chromosome 6"

Response: Thank you for this suggestion, the title was changed to "Assembly of the metapolycentromere of pea chromosome 6".

Page 6, line 161
The observation that both types of CENH3-antibodies immunoprecipitated the same sequences is interesting. Please could you indicate the similarity between both CENH3 variants?

Response: Thank you for bringing this omission to our attention; we agree that the similarity information is important for the reader. Therefore, we have expanded the text and added references to the relevant part of the Materials and Methods / CENH3 ChIP-seq analysis so that it reads as follows: "Duplicate experiments, including independent chromatin preparations, were performed for each CENH3 variant using either one antibody (P23 for CENH3-2) or two different antibodies (P22 and P43 for CENH3-1); both anti-CENH3-1 antibodies were raised against an identical peptide in rabbit (P22) and chicken (P43). The antibodies were targeted to the N-terminal tail,

which is the most variable part of the CENH3 proteins, resulting in only 35% similarity of the peptide used to raise the CENH3-2 antibody P23 to the CENH3-1 sequence, and 43% similarity of the peptide used to raise CENH3-1 antibodies P22 and P43 to the CENH3-2 sequence [6-8]."

Page 6, line 164.
'To emphasize the different centromere structure, I would suggest adding "extended". It would read: "The extended primary constriction showed…."

Response: Thank you for this suggestion, the sentence was modified accordingly.

Page 7, line 230
Just a note, the same observation regarding the linear signals of centromere repeat and linear signals across chromatids of non-centromeric satDNA was reported for some Rhynchospora species, see: http://www.ncbi.nlm.nih.gov/pubmed/27645892

Response: Thank you for this note. We are aware of these similarities, as some of us were also involved in the Rhynchospora study. We suspect that the same principles are at play in the contrasting metaphase arrangements of CENH3-associated and CENH3-free satellites in pea metapolycentromeres as in holocentric chromosomes. We hope that further study of additional cases of both types of centromeres will allow us to test this hypothesis in the future.

Page 10, line 313
Please change to:" histone H2A phosphorylation at Thr120 ((H2AT120ph) is detected across almost the entire length of holocentric chromosomes at the outer periphery of chromatids, but is restricted….

Response: Thank you for this suggestion, the sentence was modified accordingly.

About the suggested model metapolycentromere formation, a request
Please, would it be possible to prepare a schemata which would summarize the potential process of metapolycentromere formation? I think that future readers would appreciate such a summary.

Response: A new figure (Fig. 5) was added to the main text to graphically summarize the suggested model. Reference to this figure was added to Discussion, p. 11: "Our results support the view that the expansion of metapolycentromeres is facilitated mainly by the spreading of CENH3 chromatin from the centromere into adjacent chromosome arms, as depicted in Fig. 5.".

Figures
The size bars are missing in most chromosome/interphase pictures. Please add.

Response: Thank you for bringing this omission to our attention. The size bars were added to Fig. 3, Fig. 4C (all chromosomes in this composite image were taken at the same magnification), and to Supplementary data Fig. S5.

Reviewer #2: The authors describe a comprehensive assembly and annotation of a 177.6 Mb region of pea (Pisum sativum) chromosome 6, including the 81.6 Mb centromere region (CEN6) and adjacent chromosome arms, with the intent of interpreting how metapolycentromeres evolved. They provide evidence for an evolution via the expansion of centromeric chromatin into neighboring chromosomal regions with accumulation of novel satellite repeats. The assembly and annotation is

of very high quality, and the manuscript may stand alone on these merits. The assembly revealed a clear evolutionary interpretation of centromere dynamics in metapolycentric chromosomes. They report that pea metapolycentromeres are strikingly composed by different families of satellite repeats subdivided into distinct centromere domains in contrast to most commonly found single-domain repeat-based monocentromeres. Furthermore, the authors nicely describe and interpret the genomic and cytological data with well-prepared figures.

The CENH3-ChIP-seq experiments were well designed and performed, including the necessary controls and replicates.

The repeat annotation and both in silico and in situ analysis of centromere chromatin is impressive.

The oligo-painting FISH analysis presents a very clever strategy to study the evolution and expansion of metapolycentromeres.

Some comments follow below:

I am aware of a new development of the Hifiasm assembler which now integrates HiFi reads and Ultralong Nanopore reads. I wonder if the authors tried that assembly strategy on their data already and if it would make sense to compare the assemblies using this strategy, in order to remove the single gap present in CEN6.

Response: We did not attempt to perform the hybrid HiFi/Nanopore assembly because this option was not available at the time we completed our assembly. We tested it only later on another plant species and did not obtain a significant improvement over assembly with HiFi reads alone, so we did not consider reassembling our CEN6 reads. In addition, we believe that this approach would not help to eliminate the only remaining gap in the CEN6 sequence, as this gap is located in a long array of highly homogenized satellite repeats. The lack of sequence variability in the sequence regions surrounding the gap prevented its closure even with high-precision HiFi reads, and we could not manually bridge it with ultralong nanopore reads because their high error rate prevented their unambiguous mapping to this region.

The centromere effect on recombination rates of metapolycentromere compared to typical monocentromeres is remarkable. I am surprised the authors give so little detail and do not discuss these important findings. Is the centromere effect similar to monocentromeres? Is there a gradient reduction of CO rates in pericentromeric regions? Or it is rather an abrupt transition between hot and cold CO regions?

Response: The suppression of recombination is evident from Fig. S1A, which shows the positions of the genetic marker sequences in the assembly compared with their positions on the genetic map. The nonrecombining region extends over ~10 Mb from the primary constriction to the chromosome arms, and apart from the large size of the primary constriction, the recombination rate profile along CEN6 is not fundamentally different from that on some monocentric chromosomes. For this reason, and also to limit the size of the manuscript, we do not address this issue in the Discussion.

This study is interesting primarily because of the unique centromere structures of Pea chromosomes. The findings that metaplycentromere evolved rather by extension of the original monocentromere is interesting. It is unclear what sequence features if any Pea centromeric repeats

have that facilitate CENH3 deposition in comparison to non-centromeric ones. Clearly, they show similar high order organizations.

So, although much of what is presented on metapolycentromere organization was already known from previous papers from the same group, this manuscript is an excellent genome analysis of nearly T2T Pea chromosome 6. The centromere analysis included in the study is very well done and helps to understand the evolution of metapolycentromeres and could potentially be a way for the evolution of repeat-based holocentromeres. The paper would have been more complete had they carried ChIP-seq using more histone marks as well to get a more comprehensive perspective of the overall epigenetic regulation of metapolycentromeres.

Response: We thank the reviewer for these positive comments and agree that the inclusion of ChIP-seq data obtained with additional antibodies against different histone marks would complement well the presented data on sequence organization and CENH3 distribution in CEN6. However, we felt it more appropriate to defer this topic to a possible follow-up paper, in which it would receive more space than in the current paper, in which much of the space was devoted to the assembly itself. In the near future, we hope to obtain assemblies of comparable quality to CEN6 from the remaining pea centromeres and to perform the proposed epigenetic profiling in all centromeres, thus providing better data for generalization by identifying patterns common to all seven pea centromeres.

Reviewer #3: This impressive study reports characterization of metapolycentric centromeres from Pisum, which is a structure intermediate between mono- and holo-centric. Metapolycentric species show a large primary cytological constriction accounting for up to a third of the chromosome. This is a novel centromere organisation and the authors provide a detailed molecular portrait, which represents a new contribution to the field. However, there are a number of improvements I would like to see before acceptance.

The authors use long read ONT and HiFi data to generate an assembly of pea CEN6. A semi manual approach to assembly is used, in addition to automated methods, eg HiFiasm. It would be interesting to see coverage analysis of the ONT and HiFi reads aligned back to final assembly.

Response: The coverage by ONT reads is provided along with the methylation profiles in Figs. S3A-C ("coverage" tracks), as the methylation frequencies were analyzed in the nanopore reads mapped to the assembly.

In response to this reviewer comment, we have also added the ONT coverage track to the CEN6 assembly information available via online genome browser (http://w3lamc.umbr.cas.cz/lamc/jbrowse.php). We have also mapped HiFi reads to the assembly and generated corresponding track in the online genome browser (labeled "PacBio_coverage").

The paper focuses on CEN6, as it is one of the largest in the genome – however, if automated assembly has been performed I wasn't clear why the other centromeres have not been reported?

Response: The other centromeres were not reported because they could not be reliably assembled using automated assembly alone. Although for CEN6 we found that the automatically generated HiFi contigs were mostly accurate and matched the nanopore scaffolds generated by the semimanual approach, much work was still needed to scaffold and especially to verify the assembly

using FISH mapping of single-copy and repetitive probes to chromosomes at different stages of condensation, as shown in the examples in Fig. S1.

An interesting picture with respect to centromere tandem repeats is observed. There are multiple centromere located tandem repeat families, some of which are CENH3 occupied. Generally the relationships between the reported repeats, if any, are not well described. Simple histograms of repeat lengths would be useful to see. Which of the repeats are more closely related, and does this correlate with CENH3 occupancy?

We thank the reviewer for highlighting the need for more detailed information about the major families of satellite repeats present in CEN6. We did not include the details because all these satellites have been described and compared in frame of our previous studies that are cited in the manuscript (Introduction, p. 4: "...centromeres of pea are populated with a large number of different satellite repeats [6,20], ….”). See for example ref[20]: https://doi.org/10.1093/molbev/msaa090

However, to make this information easily accessible to readers of the present manuscript, we added a new supplementary Fig. S6 including dot-plot similarity comparison of CEN6 satellites that shows their monomer lengths and demonstrates that there is no sequence relationship between different families. We references to the new Fig. S6 in the Fig. 2 legend: "… Since there are no significant sequence similarities between different FabTR families (*Supporting information*, Fig. S6), their comparisons are not plotted."

One tool I would recommend be used is TRASH https://github.com/vlothec/TRASH which will allow more precise definition of tandem repeat families. It would also be interesting to define the occurrence of satellite higher order repeats, which is functionality included with this tool.

Response: As mentioned above, the definition of satellite (super)families in pea and its related species has been done before. However, we are grateful for the mention of this tool, which has escaped our attention as it is still unpublished. Unfortunately, we encountered an execution error when we ran it with our data (the bug report was submitted to the authors via github).

A further tool that may be nice to use is StainedGlass https://github.com/mrvollger/StainedGlass This would allow a single identity heat map to be made over CEN6 that could be presented with the array specific dotplots shown in Fig. 2. A StainedGlass map would provide a nice overview of sequence identity across the entire CEN6 region.

Response: Thank you for this suggestion. However, given the high sequence divergence of the CEN6 satellite families (Fig. S6), comparing them using this tool would not provide any information to show. Indeed, running StainedGlass for the entire CEN6 region yielded a blank plot highlighting only the similarities between each FabTR-10 array.

The immunocytology data in Fig 4A-B is very nice, as us Fig 3C which indicates repeat family turnover between species.

I also liked Fig. S1, particularly the clear correlation with cM data – I think this is worth considering for a main figure?

Response: Thank you. We considered this plot to be included into Fig. 1, however, it was not possible due to the space limitations.

More analysis on the gene content in the polymetacentromere region, compared to the rest of the arms would be good. It seems there are abundant genes between the CENH3 peaks. Are there are striking GO differences? Or differences in RNA-seq levels? Are these genes more heterochromatic (ie higher DNA methylation)?

Response: We did compare methylation profiles of the genes, providing this information in Fig. S3D and summarizing it in the Results (p. 6): "...gene methylation frequency histograms, which showed hypomethylation of a substantial proportion of CG and CHG motifs, and high levels of methylation in the remaining motifs, resulting in a bimodal histograms (Fig. S3D). No difference was observed between the methylation patterns of genes located within the centromere and those located in chromosome arms."

However, we believe that this information is still too limited to make general statements about patterns inside versus outside the centromere because our data represent only one of the seven pea centromeres, and especially because we do not have complete chromosome arms for comparison (for example, chromosome ends could have higher gene density and different epigenetic profiles, as reported in some other plant species). For these reasons, we believe it is more appropriate to perform analysis of GO differences and expression levels when whole genome assembly of comparable quality is available.

The transposon analysis is interesting – were any TEs found within the satellite arrays, or in areas of CENH3 enrichment?

Response: Yes, there are LTR-TE insertions in some satellite arrays, although they are not frequent. The insertions occur in both, CENH3-associated and CENH3-free satellite arrays.

The CHG methylation looks very high >80% along the chromosome – in other plants CHG would be lower eg 30-40%. The ONT based calls from Deepsignal-plant are trained on Arabidopsis and rice, which may not be the best model for pea? Due to the very high CHG levels it would be worth to compare to regular BS-seq data to confirm this finding.

Response: We agree that the >80% CHG methylation is higher compared to some plant species, but this is not an exceptional observation. In the recent study of the faba bean genome (https://doi.org/10.1101/2022.09.23.509015), Jayakodi et al. reported 88.2% of methylated cytosines in the CHG context. This high CHG methylation level was determined using enzymatic methylation sequencing, a method fundamentally similar to BS-seq. Although we cannot exclude the possibility that the methylation basecalling model used in our analysis has some bias, we believe that the same results obtained using biochemical analysis in the closely related faba bean strongly support our results from the pea genome. Thus, these high methylation levels could be taxon-dependent or a feature of large, repeat-rich plant genomes such as pea and faba bean.

Interestingly, Fig S3 (I think this also should be in the main figures) shows a very interesting difference between two TR-10 arrays that are both CENH3 occupied, but only one shows a CHG drop, as observed in Arabidopsis (Naish et al). On line 147 – please provide information on the ChIP-seq read lengths.

Response: The information about the ChIP-seq read length and their analysis is provided in the "Materials and Methods / CENH3 ChIP-seq" analysis section (p. 14): "DNA fragments …were

sequenced on the Illumina platform (Admera Health, NJ, USA) in paired-end, 150 bp mode." and "The resulting reads were quality-filtered and trimmed using Trimmomatic [56] (minimum allowed length = 100 nt)…" Thus, the mapped read sequences were 100 nt long.

Due to the repetitiveness of these sequences, what controls or efforts were made to confirm that short read alignments were accurate in these regions?

Based on the mapping output data and our visual inspection of the read mapping in the IGV browser, we found that the majority of the mapped reads were identical to the assembly over their entire length, while the rest were mapped with a few mismatches.

In the methods I note that multi mapped reads were dropped – could this create biases?

Response: No, because we actually used both approaches (multi-mapped and uniquely mapped reads) in parallel and they provided very similar enrichment profiles. In Methods, we write "Subsequent analysis was performed on full output from Bowtie2 program and on output where all multimapped reads were filtered out." - the first option (full output) provides information about multimapped reads, while the second option analyzes only the uniquely mapped reads. The detailed comparison of read mapping modes can be found for all antibodies in Fig. S2, which shows "Mapping of reads onto the assembly either in multilocus mode (A) or single-mapping mode (B)" (Fig. S2 legend).

Which ChIP-seq data is shown in Fig. S3B-C - is this unique mappers, or all mapped reads?

Response: Thank you for bringing this omission to our attention. We have amended the legend to Fig. S3 B,C to state in full: "The CENH3 ChIP-seq track shows enrichment peaks identified by the epic2 program using multi-mapped reads generated with the P22 antibody."

On line 144 – please provide more detail on the annotation methods used for these distinct sequence classes.

Response: The annotation methods are described in detail in the "Materials and Methods / Assembly annotation" section of the manuscript (pp. 13-14). Since many methods were used for the annotation, we are concerned that describing them in Results would disrupt the text flow of this section, and therefore prefer to place this information in Materials and Methods.

Line 171 – Im unclear what 'no significant effect' means here – clearly the sequences are present and so I would say they do have a significant effect – perhaps the authors mean that they are a relatively small amount of the sequence?

Response: Yes, we meant that this small number of elemental copies has "… no significant effect on centromere composition", as stated in the text.

Line 360 – 'CEN180' should be italics.

Response: Corrected.
