# Supplementary material for: Assembly of the 81.6 Mb centromere of pea chromosome 6 elucidates the structure and evolution of metapolycentric chromosomes
Source: PLoS Genet. 2023 Feb 3;19(2):e1010633. doi: 10.1371/journal.pgen.1010633 (PMC10027222; doi:10.1371/journal.pgen.1010633)

Response to the reviewers' comments and corresponding changes made to the manuscript

Reviewer #1: I'm satisfied with the revision of the manuscript and with the newly prepared figure 6 (proposed model).

Reviewer #2: The authors have addressed all my comments and concerns.

Reviewer #3: The authors have made an adequate attempt to respond to my comments, but more documentation would have been beneficial for a couple of points.

Comment: They mention having run StainedGlass, but that it yielded a blank plot. This is surprising, as they already show dotplots for the repeat arrays that should certainly be seen in a StainedGlass output. Provision of the plot from this approach in the revision document would be helpful to see even if it doesn't figure in the main manuscript.

Response: We apologize for any confusion caused by our previous response. We would like to clarify that due to the high level of sequence divergence between different satDNA families (as demonstrated in Supplementary Fig. S6), the StainedGlass analysis of the entire CEN6 assembly resulted in a plot that was largely empty, with only small regions displaying similarities within individual arrays and between the eight loci containing satellite FabTR-10. We have included the StainedGlass plot, annotated with satDNA array positions, as an attachment to this document for review. We believe that it illustrates our point that the StainedGlass plot does not provide any additional information that is not already presented in Fig. 3 with much better detail.

Comment: I still think inclusion of the cM data in the main figures would be beneficial. I wasn't aware that this journal has strict limitations on figures and my recommendation to the editor would be to allow space for this important data if there is a limitation.

Response: The requested data has been included into the main text as a new figure (Fig. 2). Figure legend: "**Fig. 2. The primary constriction coincides with a region of reduced meiotic recombination.** The graph shows the location of genetic marker sequences in the CEN6 assembly compared to their position in the linkage group LGII of the pea genetic map [22]."

Comment: The response to my question about LTR insertions in the satellite arrays is also very cursory and lacks any significant detail.

The original question and our response:

The transposon analysis is interesting – were any TEs found within the satellite arrays, or in areas of CENH3 enrichment?

Response*: Yes, there are LTR-TE insertions in some satellite arrays, although they are not frequent. The insertions occur in both, CENH3-associated and CENH3-free satellite arrays.*

Response: We apologize for the lack of detail in our previous response regarding LTR insertions in satellite arrays. Below we provide a table with detailed information on the numbers and types of mobile element insertions found within satDNA arrays. Our findings indicate that there are generally very few insertions present in the arrays, considering their extensive length of up to 2 Mbp. These small numbers of insertions make it impossible to infer eventual trends or preferences of individual element lineages regarding their target. We hope this additional information clarifies our previous response and addresses your concerns.

					Ty1/copia			Ty3/gypsy			
Satellite	array	length [bp]	LINE	LTR/ unclass	Angela	Ivana	SIRE	CRM	Tekay	Athila	Ogre
FabTR-10	1	892943	0	0	0	1	0	0	0	1	0
	2	461861	0	0	0	0	0	0	0	0	1
	3	295714	0	0	0	0	0	1	0	0	1
	4	359320	0	0	0	0	0	1	0	0	0
	5	539157	0	0	0	0	0	0	1	1	1
	6	362914	0	0	0	0	0	0	1	0	0
	7	239445	0	0	0	0	0	0	0	0	0
	8	230331	0	0	0	0	0	0	0	0	1
FabTR-48		322447	0	0	0	0	0	0	0	0	0
FabTR-49		619846	0	0	0	0	0	0	0	0	0
FabTR-50		449723	1	0	1	0	0	0	4	1	10
FabTR-85		1818553	0	1	0	0	1	0	8	0	0
FabTR-106		1974497	0	0	0	0	2	0	5	0	2
FabTR-107		1452303	0	0	0	0	0	0	2	0	6

StainedGlass output for the whole CEN6 assembly

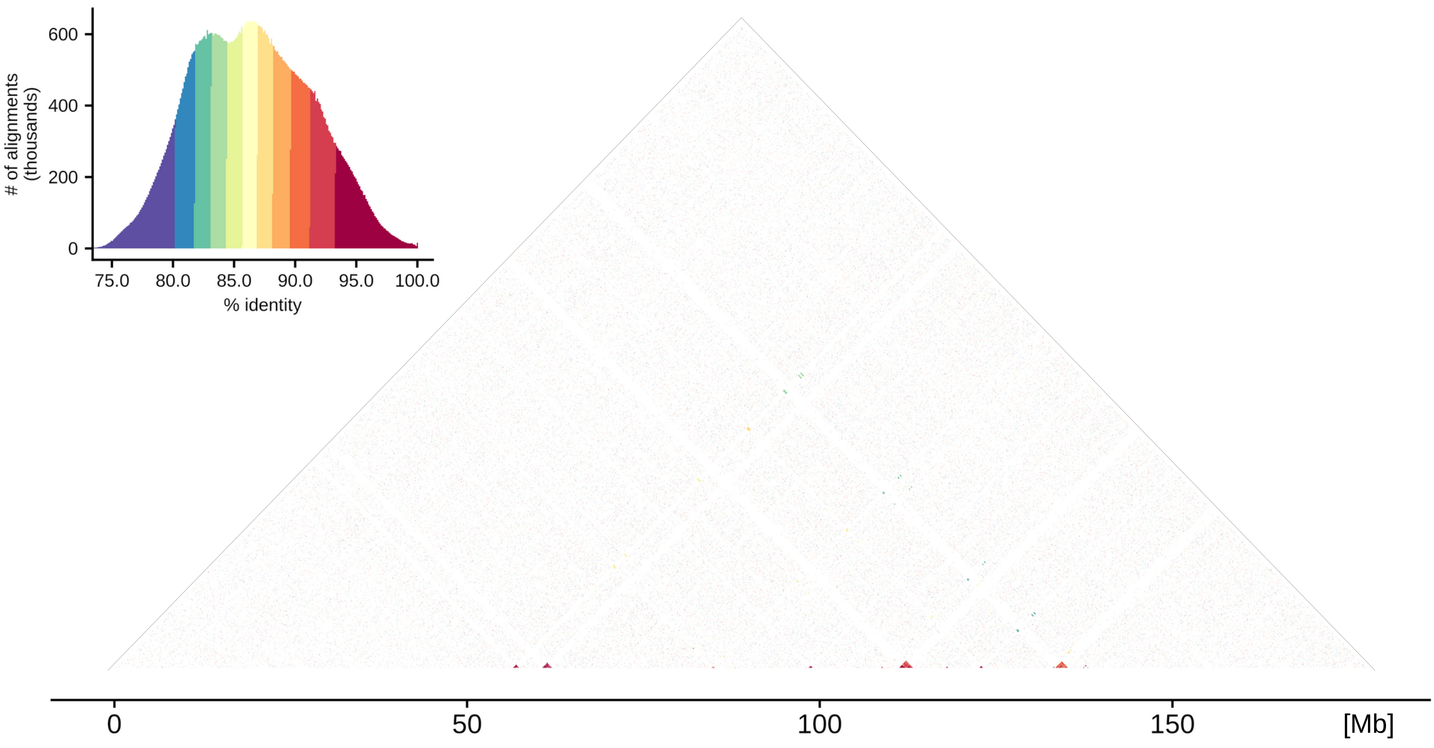

Positions of satDNA arrays

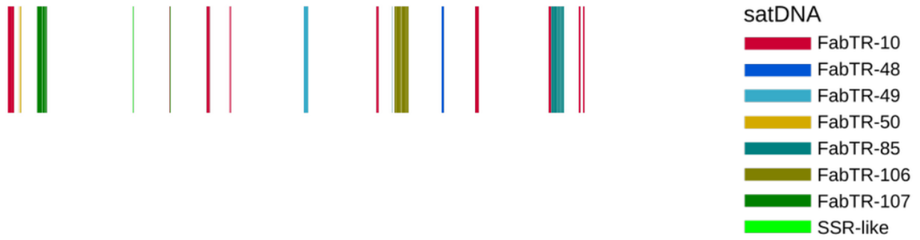

Supplement: Attachment — Submitted filename: response_REV2.pdf [file pgen.1010633.s008.pdf]
